# Supplementary material for: Resolving the full spectrum of human genome variation using Linked-Reads
Source: Genome Res. 2019 Apr;29(4):635–45. doi: 10.1101/gr.234443.118 (PMC6442396; doi:10.1101/gr.234443.118)
Supplement: Supplemental Material [file supp_gr.234443.118_Supplemental_longranger-2.2.zip › longranger-2.2.tar.bz2!/lr2.2-public/lib/stan/cmdstan/stan/doc/api/html/agrad_2rev_2functions_2falling__factorial_8hpp_source.html]

Stan: src/stan/agrad/rev/functions/falling\_factorial.hpp Source File


|  |  |
| --- | --- |
|  | Stan  2.5.0  probability, sampling & optimization |


- Main Page
- Related Pages
- Namespaces
- Classes
- Files

- File List
- File Members

All Classes Namespaces Files Functions Variables Typedefs Enumerations Enumerator Friends Macros Pages

- src
- stan
- agrad
- rev
- functions

falling\_factorial.hpp

Go to the documentation of this file.

1 #ifndef STAN\_\_AGRAD\_\_REV\_\_FUNCTIONS\_\_FALLING\_FACTORIAL\_HPP

2 #define STAN\_\_AGRAD\_\_REV\_\_FUNCTIONS\_\_FALLING\_FACTORIAL\_HPP

3

4 #include <stan/agrad/rev/var.hpp>

5 #include <stan/agrad/rev/internal/vd\_vari.hpp>

6 #include <stan/agrad/rev/internal/dv\_vari.hpp>

7 #include <stan/agrad/rev/internal/vv\_vari.hpp>

8 #include <stan/math/functions/falling\_factorial.hpp>

9 #include <boost/math/special\_functions/digamma.hpp>

10

11 namespace stan {

12  namespace agrad {

13

14  namespace {

15

16  class falling\_factorial\_vv\_vari : public op\_vv\_vari {

17  public:

18  falling\_factorial\_vv\_vari(vari\* avi, vari\* bvi) :

19  op\_vv\_vari(stan::math::falling\_factorial(avi->val\_, bvi->val\_), avi, bvi) {

20  }

21  void chain() {

22  avi\_->adj\_ += adj\_ \* stan::math::falling\_factorial(avi\_->val\_, bvi\_->val\_) \* boost::math::digamma(avi\_->val\_ + 1);

23  bvi\_->adj\_ -= adj\_ \* stan::math::falling\_factorial(avi\_->val\_, bvi\_->val\_) \* boost::math::digamma(bvi\_->val\_ + 1);

24  }

25  };

26

27  class falling\_factorial\_vd\_vari : public op\_vd\_vari {

28  public:

29  falling\_factorial\_vd\_vari(vari\* avi, double b) :

30  op\_vd\_vari(stan::math::falling\_factorial(avi->val\_, b), avi, b) {

31  }

32  void chain() {

33  avi\_->adj\_ += adj\_ \* stan::math::falling\_factorial(avi\_->val\_, bd\_) \* boost::math::digamma(avi\_->val\_ + 1);

34  }

35  };

36

37  class falling\_factorial\_dv\_vari : public op\_dv\_vari {

38  public:

39  falling\_factorial\_dv\_vari(double a, vari\* bvi) :

40  op\_dv\_vari(stan::math::falling\_factorial(a, bvi->val\_), a, bvi) {

41  }

42  void chain() {

43  bvi\_->adj\_ += adj\_ \* -stan::math::falling\_factorial(ad\_, bvi\_->val\_) \* boost::math::digamma(bvi\_->val\_ + 1);

44  }

45  };

46  }

47

48  inline var falling\_factorial(const var& a,

49  const double& b) {

50  return var(new falling\_factorial\_vd\_vari(a.vi\_, b));

51  }

52

53  inline var falling\_factorial(const var& a,

54  const var& b) {

55  return var(new falling\_factorial\_vv\_vari(a.vi\_, b.vi\_));

56  }

57

58  inline var falling\_factorial(const double& a,

59  const var& b) {

60  return var(new falling\_factorial\_dv\_vari(a, b.vi\_));

61  }

62  }

63 }

64 #endif

dv\_vari.hpp

falling\_factorial.hpp

vd\_vari.hpp

stan::agrad::var::vi\_

vari \* vi\_

Pointer to the implementation of this variable.

**Definition:** var.hpp:40

var.hpp

stan::agrad::falling\_factorial

fvar< T > falling\_factorial(const fvar< T > &x, const fvar< T > &n)

**Definition:** falling\_factorial.hpp:15

stan::agrad::var

Independent (input) and dependent (output) variables for gradients.

**Definition:** var.hpp:27

stan::agrad::digamma

fvar< T > digamma(const fvar< T > &x)

**Definition:** digamma.hpp:16

vv\_vari.hpp

stan::math::falling\_factorial

boost::math::tools::promote\_args< T1, T2 >::type falling\_factorial(const T1 x, const T2 n)

**Definition:** falling\_factorial.hpp:53


---

[ Stan Home Page ]

*© 2011–2014,
Stan Development Team.*
